# Learning to Fail Better: Reflections on the Challenges and Risks of Community-Based Participatory Mental Health Research With Inuit Youth in Nunavut

**DOI:** 10.3389/fpubh.2021.604668

**Published:** 2021-03-12

**Authors:** Polina Anang, Nora Gottlieb, Suzanne Putulik, Shelley Iguptak, Ellen Gordon

**Affiliations:** ^1^Department of Psychiatry, University of Manitoba, Winnipeg, MB, Canada; ^2^Department of Health Care Management, Berlin Technical University, Berlin, Germany; ^3^Department of Population Medicine and Health Services Research, Bielefeld University, Bielefeld, Germany; ^4^Naujaat, NU, Canada

**Keywords:** Inuit, youth, resilience, mental health promotion, community-based participatory research, engaging stakeholders, collaboration, co-production

## Abstract

Community-based participatory research (CBPR) is a mine field of moral dilemmas. Even when carefully planned for and continuously critically reflected upon, conflicts are likely to occur as part of the process. This paper illustrates the lessons learned from “Building on Strengths in Naujaat”, a resiliency initiative with the objective of promoting sense of belonging, collective efficacy, and well-being in Inuit youth. Naujaat community members over time established strong meaningful relationships with academic researchers. Youth took on the challenge of organizing community events, trips out on the land, and fundraisers. While their creativity and resourcefulness are at the heart of the initiative, this paper explores conflicts and pitfalls that accompanied it. Based on three themes – struggles in coming together as academic and community partners, the danger of perpetuating colonial power structures, and the challenges of navigating complex layers of relations within the community – we examine the dilemmas unearthed by these conflicts, including an exploration of how much we as CBPR researchers are at risk of reproducing colonial power structures. Acknowledging and addressing power imbalances, while striving for transparency, accountability, and trust, are compelling guiding principles needed to support Indigenous communities on the road toward health equity.

## Introduction

“Ever tried. Ever failed. No matter. Try again. Fail again. Fail better.” This literary quote from Samuel Beckett (1) epitomizes the current state of affairs in community-based participatory mental health promotion with Indigenous communities in Canada. Mental health among Inuit youth is considered a public health emergency of epidemic proportion, with suicide rates among the highest worldwide (2–4). The age groups hit hardest by suicide in Nunavut are 18–27 year olds, followed by 13–17 year olds. Suicide rates are 10 times higher among Inuit compared to the non-Indigenous Canadian population (5). Mental health promotion in youth has been identified as an important goal in suicide prevention in Nunavut (6). Inuit institutions and researchers have emphasized that listening to youth and involving them as partners in the design and implementation of well-being initiatives is imperative (7).

This paper reflects on lessons learnt from “Building on Strengths in Naujaat”, a community-based participatory youth suicide prevention initiative, with two main goals: to understand the pitfalls of reproducing social and health inequalities, and to contribute to moving mental health promotion in Inuit communities forward. The method chosen for this study was reflection, postulated as a valid element of social inquiry, which explicitly recognizes the socially constructed character of knowledge about human realities. “Building on Strengths in Naujaat” commenced as a collaboration between Naujaat youth and University of Manitoba researchers (8). It originated from conversations with Naujaat Health Center employees, who emphasized that improving access to mental health services was important but not impactful enough in the context of communities in mourning from so many losses, weighed down by intergenerational trauma, and by economic hardships. Naujaat Hamlet Council Elders requested that, instead of talking about “Inuit youth suicide,” the initiative shift the discourse to the role of mutual support and emotional ties within the community, intergenerational dialogue and cultural continuity in the youths' future planning: “Why do we have to talk about suicide all the time? Let's talk about love!” (Agatha Crawford, Naujaat Elder). This stance is supported by the literature. Suicide awareness campaigns were found to increase the risk of suicide becoming entrenched in cultural self-image (9), whereas cultural continuity and community cohesion have been described as protective factors (10–16).

These ideas of approaching the issue of suicide in a different way, while acknowledging the colonial context, made the academic partners consider a community-based participatory research (CBPR) approach. It was regarded as a good fit because it considers dignity, autonomy, and assets of a “community” as a frame of reference that guides research (17). The Inuit tradition of emphasizing community as opposed to the individual presents an advantage of CBPR over traditional mental health promotion measures, which focus on individual-level risk and protective factors. Individual resilience is not necessarily translated into community resilience; and community resilience is a highly dynamic process that is transformed by ever-evolving structural factors such as social, political and economic context, personal relations, and value systems (18). The emphasis on collaboration and mutual learning requires flexibility and reflective professional practice (19). This was seen as a further strength of CBPR as a framework for our project because it prevents, to a certain extent, the reproduction of colonial power relations. Instead, the collaborative process requires the Qallunaat partners to learn about Inuit values, and to acquire a better understanding of how traditional Inuit principles (Inuit Qaujimajatuqangit, IQ) are translated into decision-making and actions. Finally, CBPR focuses on the structural roots of social and health inequalities such as colonial histories, racial and patriarchal oppression, and inequalities in access to power and material resources and aims at collective capacity, efficacy and empowerment as pathways toward social change (20). In the given context, these features made CBPR the best fit for an action-oriented research undertaking.

This article renders insights into challenges and learning opportunities from “Building on Strengths in Naujaat.” To this end, it analyzes the team's reflections through the lens of three overarching themes: the need to bridge conflicting expectations and pressures of academic Qallunaat (white, non-Inuit) and Inuit community partners; the inherent danger of perpetuating colonial power structures; and the challenges of navigating complex layers of relations within the community. Through analyzing our respective narratives, experiences and thoughts from an equity perspective, the three themes are placed in a theoretical context. The purpose of this paper is to explore how much we as CBPR researchers, while intending to promote collaboration, collective efficacy, and social and health equity, are at risk of reproducing power imbalances. Our reflections are intended to help prepare teams for CBPR with marginalized populations, to support critical reflection of their roles, and to contribute to discussions among the academic community (21). Most importantly, they are intended to make space for strong Indigenous voices, and to point to the power imbalances that get in the way of health equity (22).

In section Materials and Methods we describe a brief history of the “Building on Strengths in Naujaat” initiative, including its conceptual grounding in CBPR principles, and the method of reflection. In section Results the themes illustrating conflicts and dilemmas encountered in the process of implementing the initiative are presented. In section Discussion we summarize the lessons learnt.

## Materials and Methods

### Research Setting: “Building on Strengths in Naujaat” Initiative

“Building on Strengths in Naujaat” is a youth suicide prevention initiative that came together as a response to a need formulated by community members. Young people, their parents and grandparents, nurses, and teachers expressed the pain of losing a family member, a friend, a patient, or a student to suicide and wished to contribute to a future with an emphasis on hope, creativity, and better opportunities for youth to live up to their fullest potential. This made CBPR a perfect framework for engagement. “Building on Strengths in Naujaat” involves community members and Qallunaat researchers as partners at all stages of the initiative. The goal is to build capacities,including a deeper appreciation of Inuit ways of knowing and knowledge of the Inuit concept of well-being for academic partners, as well as coping skills collective agency and efficacy for community partners, and to eventually achieve community ownership of the project.

The initiative is guided by an all female team [observed to be common in coproduction research (23)], comprised of two Inuit youth group leaders (SP & SI), and three Qallunaat researchers (PA, EG, NG). While SP and SI are at home in Naujaat, Nunavut, Canada, the Qallunaat are residents of Winnipeg, Manitoba, Canada (PA & EG), and of Leipzig, Germany (NG). Naujaat based authors are both young mothers. (SI) has an Office Administration degree from the Arctic College and is employed by the Health Center as Community Oral Health Coordinator. (SP) has past experience as a volunteer firefighter and is currently working for the Co-op gas station. The first author (PA) works in Naujaat for 6 weeks annually in a clinical capacity as a child and adolescent psychiatrist. Overall she has spent over 200 days in the community since 2014. The senior author (EG) has visited Naujaat twice for a week each visit, and together with PA provides clinical services as a family therapist to the community via telehealth on an ongoing basis. Second author (NG) is a Public Health researcher with experience engaging marginalized populations in research in different contexts. Having come up with the initial idea for a CBPR approach, she continues to provide a critical perspective on equity and the structural determinants of health. Her geographical distance proved indispensable for questioning what we otherwise tended to take for granted, but one of the authors (SP) saw this as an obstacle to her ability to fully comprehend the reality on the ground in Nunavut.

The academic partners (PA, NG, EG) formulated the written portion of this paper; however all ideas and lessons learned were co-produced and reviewed in oral discussions with the community co-authors (SP, SI). Naujaat based co-authors requested to be the voices of the youth group without standing out. We agreed to avoid mentioning their opinion directly, as it was perceived as possibly threatening future employability at the Health Center, as well as reigniting family conflicts. Other members of the youth group who contributed to our discussions chose not to go on record out of similar concerns.

The idea for this study was formulated in dialogue with Naujaat Hamlet Council, Naujaat Health Committee, and Naujaat Elders. It was repeatedly stated by Elders that looking at the positives, at strengths and hopes, is crucial for building resilience. We outlined that the academic partners could help by supporting the youth in developing their visions of a healthy community. Our target population were young people residing in Naujaat between the ages of 16–25 years. Their wishes and ideas received a central role in the design of the study as the starting point of all project activities. Inuit youth were designated as co-creators of the action plan. Collaboratively, community and academic partners chose activities, applied for funding, executed them, and reflected on the benefits to the individual, the family, and the community. The ultimate goal of this youth suicide prevention initiative was to co-produce a sense of agency and ownership that will promote Inuit self-determination and well-being, in accordance with National Inuit Strategy on Research (24).

To illuminate the wishes, hopes, and dreams of the youth of Naujaat as a first step, the project started out with a series of six **focus groups** in April 2017. Two of the authors (SP & SI) helped to shape the questions for the focus groups, recruited participants and co-led the focus groups together with our colleague Dr. Maria Bronson, PA and EG. Themes that emerged from these groups were cultural identity and pride, sense of belonging, plans and visions for the future. To refine and consolidate these themes we conducted 12 **individual interviews** with group members. (A detailed description of the methodology and findings from the focus groups and interviews will be reported elsewhere.).

After finishing the focus groups, weekly youth group meetings developed; they built on the themes to co-produce interventions. Initially, a Qallunaat high-school teacher helped organize weekly meetings. After she left the community, the meetings stopped for several months, and resumed after a new teacher took on the organizing task. The reasons for a designated organizer were multilayered. Initially, the meetings took place in the Public Health Room of the Health Center, and the nurse in charge insisted on an “adult” (unspoken but implied: “Qallunaat”) supervisor. Later, the meetings took place at the Tusarvik Elementary School, and a teacher was needed for supervision. Additional reasons for a designated organizer were capacities to contact group members, buy snacks, and help with provisions for trips out on the land. As youth juggle responsibilities of school, work, raising children (nieces, nephews, and siblings in addition to their own babies) and helping family Elders, this coordinating role was more manageable for a volunteer teacher.

The “Building on Strengths in Naujaat” youth group organized activities that included various sports tournaments, fundraisers, sewing circles, a presentation series, and trips out on the land (ranging from day trips to three night camping trips). One of the authors (SP) was in charge of an athletic committee that applied for Tusarvik gym use, recruited community members, appointed referees, and announced rules and awards for the spontaneously built teams. Soccer, floor hockey and volleyball tournaments were put together. All teams were passionate and the audiences cheered; fun was the ultimate measure of success. Other committees took on organizing fundraising bake sales, penny sales, and sewing circles.

The most exciting activity proved to be **trips out on the land**, with staff of the community health care center. Youth group members who were otherwise hard to engage took on leadership roles. Academic partners and health staff needed guidance every step of the way. Youth decided what provisions to take, who to hire as guides, and how much gas was needed. On the way, stories were told about other trips, challenges, tragedies, and legends. Youth who were branded as trouble-makers in the community flourished while camping. They looked out for others, fetched water for the kettle, and refilled gas in the common stove. In the non-clinical setting clinical measures for protective and risk factors were replaced by a sense of purpose, a sense of belonging, and group cohesiveness. Seeing resilience in action transformed our partnership. Experiencing and sharing valuable moments became a fundamental goal of our collaboration.

The COVID-19 pandemic suspended all group activities. Youth group meetings, interviews and collection of qualitative outcome measures will resume after the pandemic restrictions have been lifted.

### Research Method: Reflection

This paper reflects on the experiences, challenges and insights of the “Building on Strengths in Naujaat” team. To this end it draws on ongoing reflection and evaluation processes that accompanied the project's implementation. These include the research team's observations and discussions, and informal unrecorded conversations with youth, their parents, and grandparents, Hamlet Council, Elders, Hamlet administrators, as well as Non-Inuit teachers, and nurses, who resided in the community between 2017 and 2020.

Reflection has been described as an important mode of learning in action-oriented and community-engaging types of research. It figures centrally in cyclical processes of “(1) action, (2) concrete experience, (3) reflective observation, (4) abstract conceptualization, and new action” (25). Critical reflection as a valid element of social inquiry is thus closely related to a hermeneutic-dialectic epistemology, which explicitly recognizes the socially constructed character of knowledge about human interactions and realities. This, in turn, requires coproduction researchers to gain awareness of their own norms and perspectives, their embeddedness in their political, socio-economic, racial, cultural and gendered position, and to question entrenched patterns of reactions and behaviors – including the framing of problems and research questions, the design and implementation of data collection, and the execution of analyses and interpretation (26). Robertson suggests that in this process the researchers' reflections and their contextualization serve a triple goal of contributing to theory development, gaining practical insights that will help improve practice, and facilitating emancipatory awareness and action (27).

Minkler (21) characterizes CBPR with marginalized populations as a “challenging but highly promising approach.” Her discussion of power imbalances and ethical dilemmas addresses racism, tensions between insiders and outsiders and conflicting interests within a community. Introspection, transparency, and open dialogue emerge as foundational guidelines for future CBPR projects (21). Mindful engagement in the context of careful consideration of costs and benefits and more reflective acknowledgment of unavoidable ethical conflicts in co-production can create novel and unexpected results. Joint decision-making by researchers and stakeholders can produce exciting outcomes when executed with proper care and reflection (23). The self-reflective process is essential for understanding the impact of power, identity, and positionality in CBPR. It helps to achieve team cohesion and is integral in making sure the research is not re-colonizing the population that makes itself vulnerable by exploring complexities (22).

In line with this concept of reflection, the “Building on Strengths in Naujaat” team has retrospectively collected pertinent episodes in the project implementation process, identified key challenges, conflicts and learning moments, and discussed related cognitive and emotional experiences. The discussions led to a mapping of the different episodes and experiences around three themes: conflicting values, expectations and pressures of academic and community partners; the dangers of reproducing colonial power structures; and the challenges of navigating complex layers of relations within the community. These three themes posed challenges for our collaboration and at the same time taught us valuable lessons about the role of relationship building as the foundation of CBPR with marginalized populations.

## Results

The following section will elaborate on these three themes and ultimately relate them to the overarching question of how CBPR can avoid reproducing colonial power structures, and instead address (current and historic) conflicts, faults and failures to live up to its promise of contributing to greater equity.

### First Theme: Struggles in Coming Together as Academic and Community Partners

The world of academia and the world of our community partners march to very different beats (28). Throughout the initiative, differences between the two worlds became manifest in various ways and posed challenges to the academic and community partners in genuinely coming and working together. In the following, we will relate three key differences: different timelines, different values, and different expectations from the initiative.

In the early phase of this project, senior researchers attempted to dissuade us from embarking on this journey by pointing out that the time investment will not pay off in terms of publications. Their prediction was accurate. Five years later, this is our second paper. Most of the academic partners' time is invested in relationship building and re-building amidst changing composition and context of the youth group, and the management of conflicts and logistic challenges. Extracting publishable results is a painstakingly slow process, when it happens at all.

All the while, the academic partners were still going too fast for the community partners. In the clinical milieu, the question “How long have you been coming to Naujaat?” is commonly asked of psychiatrists. For some patients the right answer is “three years”, and others do not view outsiders as trustworthy, even after 8 years. Youth and academic partners spent time together, shared snacks and Caribou burgers, while jointly formulating interview guidelines. Nonetheless, the answers given in both individual interviews and focus groups were mostly safe, indicating that the youth perceived the process as rushed. We realized that more consistent and reliable relationships were required to ask deeper questions. According to Attachment Theory, exploration starts after a secure attachment has been established. A pattern of consistent reliable interactions in tune with the needs of the individual will generate a safe haven from which exploration can be launched (29).

In order to get permission to “dig deeper” into the youths' dreams and visions for a better future, we had to figure out how to visit the community; that is, how to be present, attend to, and take part in ordinary life. Visiting is an essential part of community cohesiveness in Naujaat. The doors are unlocked, no one is expected to knock or ring a bell. Coming over does not require a purpose, you can just come to spend time together. Being together does not require talking. People are comfortable with silence. We also had to learn how to present ourselves at these occasions: whether and how to show vulnerability, humility, and helplessness. When one of the authors (PA) brought her daughter to attend first the Tusarvik Elementary School and later Tuugaalik Junior High, it helped her to connect in many different ways as a parent and as a non-professional human being. Leaving behind the Qallunaat attributes of talking fast and talking too much, finishing other people's sentences, and having the answer for every question, proved to be very beneficial. It is through slowing down and refocusing our efforts from collecting “useful data” to getting to know each other, that the diversity of opinions, the complexity of intergenerational respect and estrangement, and the dynamic systems of working within the colonial institutions and resisting their uniformity came to light.

Differences in values (unconsciously) held by the academic partners and the community threatened to undermine the initiative before it had even started. The academic partners approached the Hamlet Council to help identify youth with leadership potential in order to begin recruiting participants for the youth group. To their great surprise, this request was turned down, even though the Hamlet voiced unequivocal support of the initiative. As it turned out, “leadership” is not a universally positive concept. For the Inuit community the concept of leadership implies “singling out” individuals and thus runs counter to their values of equity, cohesion, and mutual help. Following consultations with Qallunaat high school teachers, the academic partners came up with a draft list of names (including the co-authors' names SP & SI); and the Hamlet Council gave its approval without hesitations.

In addition to addressing the discrepancies between academic timelines and Inuit etiquette, and between Western and Inuit values, another challenge stemmed from the partners' divergent expectations and pressures. From the initiative's beginning, the academic partners endeavored to generate funding in order to ensure the initiative's continuation. However, as with many participatory projects, securing sustainable funding proved extremely tedious, with the lack of precise outcome measures being the main reason for rejection of grant proposals. Presetting outcome measures, however, contradicted the Inuit youth's legitimate demand for an opportunity to their own visions and plans. And while the academic partners fully supported a process where the community partners would identify their path toward a better understanding of Inuit resiliency and well-being – the pressure remains to ensure the initiative's sustainable funding within existing funding structures.

Similarly, academic and community partners expect different outputs from the initiative. Academic partners need publishable research results, which are, however, abstract and irrelevant for the community partners (unless they provide travel opportunities like conference presentations). For the members of the youth group, in the short run, the excitement of going out on the land as a group, the joy of being in charge, smiles and happiness of braving the cold on the qamutiik (large traditional sled), were important signifiers of success. In the long run, SP and SI expect concrete results on the ground. The initiative will eventually be measured by the job opportunities and recreational resources created, training courses completed and translated into respectable jobs, and housing crises resolved. Anything less than that will be viewed as a let down.

### Second Theme: the Danger of Perpetuating Colonial Power Structures by Embedding the Project in Existing Infrastructure

At different stages of the initiative, the academic partners felt that both individual-level factors (e.g., socio-economic and professional status) and socio-political context made them tread a thin line between guiding the initiative and facilitating youth agency. Within that area of tension, the second theme reflects on a confrontation that ensued around issues of space and agency. Initially, “Building on Strengths in Naujaat” used the large Public Health Room in the newly constructed Naujaat Health Center. However, with new Health Center administration, the group no longer had access to the Health Center. For some time, meetings were held at a private residence of an Elder, which was problematic, because not everyone was comfortable entering the home. When the regional health authority representative offered to provide space for the group, it seemed a plausible solution at first. The youth group was offered a chance to embed its activities in existing health service infrastructure. This would also mean that participant recruitment and the scope of activities would be determined as part of a collaborative effort of the group and the health services.

The group's integration into existing health service infrastructure could have helped to resolve material challenges for the group. However, both the academic partners and the youth each had a separate set of concerns. The youth noted that the space on offer was not a safe space. They were also wary of aggressive recruitment of new group members from part of the health staff, as this would jeopardize the sense of safety within the group that had been created over time. Nonetheless, the youth were undecided whether to accept the offer.

The academic partners perceived the health services' offer as a risk of institutionalized takeover. Their main concern was that the (non-Inuit) health staff would not pay attention to the needs of the group and would impose a different structure and set of goals. From the academic partners' point of view, the initiative's main goal at that stage was for the group to learn to be in charge of all activities. The concept of enhancing a sense of agency in the young people appeared to be at risk of being sacrificed for convenience and structural support.

The academic partners eventually “protected” the youths' autonomy and ownership of the project and declined the offer on behalf of the group. As much as the youth group explicitly agreed with the decision, this step raised complex ethical questions ultimately related to colonial legacy of the health care system (30, 31). Yet, by distancing the group from the opportunity to be embedded in institutionalized hierarchies, the academic partners took a paternalistic approach potentially disregarding the capacity of the Inuit youth to resist, to resolve the dispute with administrators, and to reform existing structures. While the academic partners felt supported by SP and SI in this decision, it is hard not to see the parallel in the paternalism of both approaches.

### Third Theme: the Challenges of Navigating Complex Layers of Relations Within the Community

The third theme includes disagreement with community Elders over the distribution of project resources. A main goal of the initiative is that the youth group lead processes of decision-making each step of the way. They are encouraged to come up with ideas for activities, plan and implement them. By this token, among other activities, the youth organized sewing circles that would bring together Elders (as sewing instructors) and youth. Sewing circle planning included details of which materials to purchase, and which Elders would accompany the sewing circles. Hours and hours were spent on debating the details of fur trim, lace, warm lining, and zippers. The anticipation was mounting, everyone was looking forward to a week of creating new jappas (fur-trimmed winter jackets). Yet, without warning, anticipation turned into a whirlwind of frightened phone calls from youth and teachers to the academic partners, a perfect storm of misunderstandings. It turned out that several community Elders who were included in the planning from the start, and strongly supported giving youth autonomy and promoting agency in youth, expected to be given the project funds along with the autonomy to distribute them based on their personal preferences. While this was inconceivable for the academic partners, every youth group member explained how important it is to listen and not to contradict Elders.

This conflict was emblematic of the dual loyalty of Naujaat youth. Respecting Elders is one of the cornerstones of Inuit identity. In addition to the lived experience that would help people learn to survive out on the land, close to 75% of Inuit parents and grandparents are first or second generation residential school survivors. They are revered by the youth not only for their resilience, courage, and overcoming trauma, but also as keepers of the past who have learned how to deal with the present. Against this backdrop, it was not surprising that the group members capitulated and stated that they would be fine with giving away all the resources allocated to the sewing circle to the Elders. We were facing the prospect of antagonizing a group of Elders, with potentially devastating consequences for our standing in the community. The alternative, appeasing the Elders, would come at a cost to the integrity of the group process, not to mention risking the transparency of the funding distribution.

One of the authors (PA) was chosen to talk to the Elder she had the strongest relationship with, to clarify the misunderstanding, and explain that detailed plans were debated by the youth group and why it would be crucial to the spirit of youth empowerment to execute them accordingly. Strong emphasis was put on the fact that we must follow the protocol submitted to the funding authority and therefore this was the only action permissible within the funding mandate. The conversation was heated, albeit respectful. The sewing circles moved ahead as planned by the youth group and were a huge success, with the participation and support of Elders who were not involved in the dispute.

In this particular incident, the goal of empowering Inuit youth to make their own decisions collided with the more culturally rooted expectation of not contradicting Elders. Insights into the origins of this disagreement, how it unfolded, and its sequalae are an invaluable experience of what youth are reporting as complex negotiations between tradition (“Never talk back to an Elder.”) and fast-moving renewal of cultural identity (“My commitment to the group/my workplace/my own future requires me to contradict.”). Being caught between “old” and “new” rules, integrating IQ principles into workplace commitments, educational aspirations, and family planning is hard. This ambivalence offers a fertile ground for emotional blackmail (“If you leave the community, you are no longer my grandchild”), threats (“If you don't let me collect your paycheck…”), and alienation that has been commonly associated with families of residential school survivors (32). The importance of promoting intergenerational dialogue amidst these tensions has been pointed out to us by the Hamlet Council at the onset of “Building on Strengths in Naujaat” and remains one of the main pathways for our future endeavors.

## Discussion

This article reflects on the risks of reproducing inequalities through CBPR, the researchers' best intentions notwithstanding. It does so through the exploration of three themes, which describe challenges encountered in the process of “Building on Strengths in Naujaat”, a participatory suicide prevention initiative with Inuit youth.

The first theme puts the project's lengthy and non-linear development in the context of conflicting demands of academic funding and career advancement on the one hand, and longitudinal relationship-building in communities on the other hand. University career and funding structures promote research that yields pre-defined and immediate results; whereas Inuit etiquette values spending time together, listening respectfully, watching, and participating. It takes years to understand the diversity of voices and the significance of connections to family and land, and to engage in dignified creation of trust. This painstaking process is unattractive for funders; and it does not pay off in academic credit. If ignored, however, the research process is liable to miss its target and leave communities with a (legitimate) sense of exploitation (33).

Academic communities could help to diminish the tensions between academic and communities' demands. If universities, funding agencies, and scientific journals genuinely believe that community participation and diversity (for instance, in views, experiences, and kinds of knowledge) are valuable assets for science, better accommodation for participatory research and for a plurality of voices ought to be built into the design of career pathways, stipends, grants, and publications. At the same time, we also call on the participatory research community to step up efforts to facilitate the inclusion of participatory research in mainstream academia. Among other contributions, co-production researchers can act as reviewers for grant proposals and manuscripts; and they can formulate frameworks to help unfamiliar reviewers, editors, and readers assess the quality and outcomes of participatory research initiatives. The question of making academic findings relevant to the community remains the core task of the co-production process.

The first theme furthermore draws attention to CBPR researchers' need to be able to step out of themselves and critically reflect on the basic values that inform their actions (34). This first (near-)failure of our initiative reminded us of the importance of cultural humility and “two-eyed seeing.” Referring to Indigenous and Western points of view, the concepts of cultural humility and “two-eyed seeing” imply an acknowledgment that all perspectives and values are context-specific and therefore limited (35). While the community partners practice two-eyed seeing on a daily basis, the academic partners had to realize their limitations and need for further learning.

The second theme points out a “parallel process” of reproducing colonial protectionalism while fighting a colonial approach to institutionalization. “Building on Strength in Naujaat” has hitherto missed the opportunity to engage the team in a critical exploration of the colonial legacy of the health care system (36). In hindsight, the challenges and conflicts arising from the initiative's integration in the health services would have provided an opportunity to better understand current power structures in access to and provision of health care, and to explore youths' perspectives on the purely non-Inuit medical staffing of the Health Center, dismantled local midwifery, and mental health interventions with little regard for local cultural values. Hence, in retrospect, we ought to have more trust in our community partners' abilities to work within hierarchical institutions and in their willingness to dare, struggle, and fail rather than presume their need of protection. Given that trust, we, the team, could have seized the conflict as an opening of a dialogue with the health services, and furthermore, as a vehicle for transformation. This process in itself can be understood as promoting mental health and resiliency.

The third theme illustrates that Elders who welcomed our vision of enhancing the capacity and agency in young people at the same time expected us to adhere to the traditional way of respecting Elders. Accommodating the Elders' wishes would have produced resentment among the youth, with potentially destructive effects on the group process. This conflict caused major disruption, injury and pain for all partners involved. It showed us the limits of collaboration, dealt a blow to a strong collaborative spirit that included our relationships with the Elders, and their participation in the genesis of the group. Moreover, it strained familial ties for some of the youth. The conflict epitomizes the complexity of being embedded in a community with strong cultural values that pose irresolvable dilemmas when faced with Western norms. According to Harari (37), contradictions are inherent within every human culture, and propel us to change. The dialectic of being rooted in Inuit Qaujimajatuqangit (“What Inuit always have known to be true”) on the one hand, and on the other hand needing to adapt to modern day challenges is ongoing for young people in Naujaat. Tensions are amplified by the rigidity of rules passed on through generations, by very recent cultural genocide, and by additional stressors related to the direct aftermath of colonization such as food insecurity, overcrowded housing, high unemployment rates, lack of vocational and postsecondary education options in the community. For the academic partners, the take-home message from this theme is that we must develop a sense for the depth and emotional intensity of the dilemmas that CBPR projects can inflict on community partners, so we can empathically support each other through inevitable heart-breaking conflicts.

The limitations of this reflection paper include the imbalance of academic perspective receiving more attention than the community co-authors' perspective. Spending less time together as a team due to the COVID-19 pandemic contributed to this imbalance. We wholeheartedly support Alethea Arnaquq-Baril's proclamation that all research in Inuit Nunangat (Inuit Homeland) should be led by Inuit, should be relevant to Inuit, and should benefit Inuit communities (38). Her statement echoes Inuit Tapiriit Kanatami (ITK) calls to Inuit governance in research, aligning funding with Inuit priorities in research, Inuit ownership of data, and increasing capacity in Inuit Nunangat research (24). There is no doubt that a clear articulation of Inuit Nunangat research priorities, sustainable funding for research relevant for Inuit communities, investing in broadband access, and most importantly “Partner[ships] with governments and research institutions to develop Inuit-specific training and education programs to foster future generations of Inuit researchers” (24) will be incredibly helpful to future CBPR initiatives. Current examples of such partnerships pave the way to Inuit leadership in research (39). Qaujigiartiit Health Research Center in Iqaluit provides resources and networking opportunities for Indigenous scholars and allies working with circumpolar communities. One of the goals of NISR is the establishment of Inuit Nunangat University. Our partnership with Naujaat youth would be much more balanced if community partners could earn University credits for the work on this project while using Inuktitut and being evaluated in accordance with IQ principles, acknowledging oral contributions, activitiesandcommunity commitments (SI).

We continue to believe that CBPR provides an important framework for mental health collaboration and suicide prevention in Inuit Nunangat. Following lessons will help guide theory and practice in the future:

- Paying attention to ongoing relationships on the ground (SP).
- Continuous presence in the community furthers the development of relationships (SI).
- Critical introspection/reflection on values is a vital aspect of the CBPR process (NG).
- Ongoing discussion among team members on expectations and pressures as they arise is crucial.
- Conference and workshop travel is an enriching and eye-opening experience that would otherwise not be available to community members (SP).
- Awareness of historic and current conflicts with institutions, and how they may affect the work in the community is necessary (PA, EG).
- Ongoing recruitment of new members is important in that it allows for growth and development of the youth group (SP, SI).

Overall, “Building on Strengths in Naujaat” taught us that co-production with community partners is a worthwhile yet tenuous balance between a mutually rewarding collaboration and potential damage to the community and to research integrity; between promoting change and undermining community values; and between mobilizing resources and reinforcing inequalities. The promotion and development of mental health in Inuit Nunangat must take into account these opposing forces. If we don't pay attention to the dilemmas created by colonization and traditional values, we run the risk of reinforcing dysfunctional patterns. “Building on Strengths in Naujaat” was established with the objective of providing a group of Inuit youth with the experience of agency and ownership of the initiative. This was to promote a sense of belonging, resiliency, collective efficacy, and ultimately well-being. Framed by the wider context of colonization, issues of conflicting demands, autonomy from existing power and community structures, and cultural values in relationships with community members all impact the initiative's process and outcomes. However, from our experience, acknowledging the mine field of power imbalances while openly addressing current and historic faults and failures, provides learning opportunities that can help make Indigenous mental health collaborative research a more effective resource to support communities on their meandering path toward greater health equity.

## Data Availability Statement

All relevant data is contained within the article. To protect the confidentiality of our community partners, no further data will be made available.

## Ethics Statement

The studies involving human participants were reviewed and approved by (1) Human Research Ethics Board, University of Manitoba (2) Nunavut Research Institute. Written informed consent for participation was not required for this study in accordance with the national legislation and the institutional requirements.

## Author Contributions

All authors contributed to the conception, formulation, and revisions of this article.

## Conflict of Interest

The authors declare that the research was conducted in the absence of any commercial or financial relationships that could be construed as a potential conflict of interest.
